# Budgeting challenges on the path towards universal health coverage: the case of Benin

**DOI:** 10.1186/s13561-020-00286-9

**Published:** 2020-09-05

**Authors:** Elisabeth Paul, N’koué Emmanuel Sambiéni, Jean-Pierre Wangbe, Fabienne Fecher, Marc Bourgeois

**Affiliations:** 1 Université libre de Bruxelles, Campus Erasme, Route de Lennik 808, CP 594, 1070 Bruxelles, Belgium; 2 University of Liège, Tax Institute, Quartier Agora, Bat. B3, 4000 Liège, Belgium; 3 Faculty of Letters, Arts, and Human Sciences, University of Parakou, Parakou, Benin; 4 Lawyer, Auditor, Centre for Research and Study in Law and Judicial Institutions (CREDIJ), University of Abomey-Calavi, Abomey-Calavi, Benin

**Keywords:** Universal health coverage, Health financing, Budgeting, Public expenditure management, Strategic purchasing, Benin

## Abstract

**Background:**

In its pursuance of universal health coverage (UHC), the government of Benin is piloting a project of mandatory social insurance for health entitled “ARCH”.

**Methods:**

We analysed budget data and ARCH documents, and conducted four observation missions in Benin between March 2018 and January 2020. Results are presented in terms of the three classical objectives of public expenditure management.

**Results:**

The government of Benin faces important budgeting challenges when it comes to implementing the ARCH social insurance project: (i) the fiscal space is quite limited, there is a limited potential for new taxes and these may not benefit the ARCH funding, hence the need to prioritise fiscal resources without jeopardising other areas; (ii) the purchasing of health services should be more strategic so as to increase allocative efficiency and equity; (iii) the efficiency of the expenditure process needs to be improved, and more autonomy needs to be devoted to the operational level, so as to ensure that health facilities are reimbursed in a timely fashion in order to meet insured people’s health costs, in such a way as to avoid jeopardizing the financial equilibrium of these facilities.

**Conclusion:**

The important budgeting challenges faced by Benin when it comes to implementing its UHC policy are also faced by many other African countries. It is important to avoid a situation in which the resources dedicated by the government to the social health insurance system are at the expense of a reduction in the financing of preventive and promotional primary healthcare services.

## Introduction

Like many other African countries, Benin is confronted with important problems with regard to accessibility to healthcare. According to the recent Global Monitoring Report on universal health coverage (UHC), the service coverage index (SDG-UHC indicator 3.8.1) was at 39.6% in 2017 (down from 40.2% in 2015). Data on the incidence of catastrophic expenditure (SDG-UHC indicator 3.8.2) date back to 2011, when it was 10.9% (respectively 5.4%) and 10% (respectively 25%) of household total consumption or income [1]. More recent survey and census data indicate that only 8.4% of the population is covered by some form of health insurance. While about 40% of the population is poor, more than half of them (23% of the total population) are extremely poor [2].

Currently, the health financing system in Benin is extremely fragmented. Several financial protection schemes coexist – in particular, contributory schemes covering civil servants, retirees and employees of the formal sector; targeted fee exemptions; and voluntary community-based health insurance [3]. Health insurance is not compulsory, but this should change in the near future. In its pursuance of UHC, the President of Benin [elected in 2016] and its government launched two major reforms: on the one hand, an ambitious reform of the “supply-side” (service provision) of the health sector and its governance – including the creation of new regulation authorities overseeing the Ministry of Health, and a reinforcement of public-private partnerships [4]. A new autonomous agency in charge of planning, coordinating and implementing the national policy with regards to primary health care has been created in October 2019 [5], and other counselling and regulating bodies in charge of the hospital and pharmaceutical policies have also been created in late 2019. On the other hand, on the “demand-side” (financial protection), the government has opted for developing mandatory social insurance for health, coupled with other insurance-type social protection schemes targeting the poor and the informal sector, under the project entitled *Assurance pour le Renforcement du Capital Humain* (ARCH). The two reforms were priorities of the electoral programme of the President, the Hon. Patrice Talon, who closely monitors its design work. No such ambitious reform has been previously initiated in this sector, and the undergoing dynamic is remarkable. However, when the government shared the proposed reform agenda with stakeholders outside of the two commissions, it faced some resistance by some constituencies, and a strike action was launched in the health sector.

The ARCH project is currently piloted by a management project unit under supervision of a national steering committee [6]. The pilot implementation of the health insurance scheme is partly delegated to an autonomous agency of the Ministry of Health (*Agence nationale de l’assurance-maladie*). However, will later be managed by another agency, the *Agence Nationale de Protection Sociale*, created in 2019 under the supervision of the Ministry of Social Affairs and Microfinance, which will be responsible for the operational management and general supervision of the ARCH scheme when it is scaled up [2, 7]. A method for identifying the poorest households has been validated at the national level, and relies on a mixed approach combining community identification and a proxy means test [8]. A list of poor and extremely poor households has been pre-identified through community identification. This comprises 467,621 poor households, of which 224,213 are deemed to be extremely poor, out of a total of 1,960,615 households. The government intends to finance 100% of the health insurance premiums for the poorest (estimated 1,895,810 beneficiaries), and 40% of the premiums for the other (non-extreme) poor (estimated 2,468,254 beneficiaries) [2]. Following two actuarial studies, the health insurance annual premium’s cost (6000 CFA francs or about 10 US dollars (USD)) was finally roughly estimated on the assumption that insured people would have two medical contacts per year, times the average consultation cost at primary healthcare level. It is planned that a private sector insurance company will be given a mandate to manage the health insurance component of the ARCH [2]. Nevertheless, not all elements of the ARCH projects are set in stone as yet, because the project has only been piloted in three health districts starting in August 2019.

**Fig. 1:**
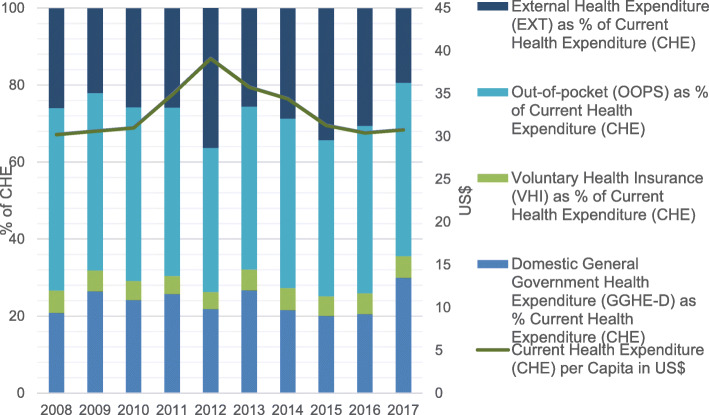
Key health financing indicators, Benin, 2008–2017. Global Health Observatory Data Repository [http://apps.who.int/nha/database/ViewData/Indicators/en ], consulted 23 December 2019

An analysis of the health financing indicators in Benin, some of which are presented in Fig. 1 below, raises several concerns. First, the total expenditure on health is low, below the threshold of 5 % of gross domestic product (GDP) commonly recognised as a minimum for UHC [9]. Health capital expenditure is particularly low (0.25% of GDP in 2015) and the health sector receives a low share of general government expenditure (three to 4 % in recent years). Second, the share of domestic private health expenditure is still significant (almost 50% of current health expenditure) and, in particular, out-of-pocket expenditure comprise more than 40% of current health expenditure, amounting to USD 38.08 (purchasing power parity) per capita in 2017. Third, the share of voluntary health insurance is very limited and stagnant over time (slightly above 5 % of current health expenditure). Finally, Benin is very dependent on development aid, since external health expenditure comprises a significant and growing share of current health expenditure (from 25% in 2007 to 30% in 2016).

The design and implementation of such an important reform as the ARCH project raises important trade-offs and challenges. This paper intends to analyse the budgeting challenges raised – both at the revenue mobilisation and at the spending levels – by the launch of the state-subsidised mandatory social health insurance in Benin. It limits itself to a consideration of the policy design and institutional challenges, without considering future implementation issues. We believe this can support reflection on the part of the Beninese authorities, but also on the part of other countries in Africa, since these challenges are faced by many governments seeking to achieve UHC.

## Methods

This paper rests mostly on the analysis of budget data *(“Lois de Finances”)*, legal acts and ARCH documents collected in Cotonou. However, we also collected information on stakeholders’ perceptions about the reform through two research projects which have followed up the development of the UHC policy in Benin since 2015. In this context, the non-Beninese authors conducted four missions in Benin in March 2018, April and October–November 2019, and January 2020, while the Beninese authors have followed the policymaking process continuously. In total, we conducted semi-formal interview with 29 persons (five of them were interviewed twice) comprising representatives of the government, development partners, private insurers and other resource persons. The informants were chosen in a reasoned manner on the basis of their technical, administrative or political skills and position (directors, managers, programme officers, technical agents) in the various institutions involved in policymaking and implementation of the social protection and health sectors. This enabled to diversify viewpoints between institutions and between informants’ positions. The semi-structured interviews aimed to collect factual data and to investigate informants’ knowledge and perceptions with regards to the ARCH project, their own intervention in the field, and their opinions as for the future perspectives of financing, coverage and policy effects. The questions were fairly open and anchored on the interventions of each stakeholder. The results from our analysis are presented in terms of the three classical objectives or functions of public expenditure management [10].

## Results

### From aggregate fiscal discipline to fiscal space for health

Aggregate fiscal discipline is a desired public sector outcome stemming from the appropriate control of public resources [10]. Whereas the term “discipline” may be viewed negatively, as a coercive means aimed at shrinking public (social) expenditure, this principle has actually been reinvigorated through the acceptance of a more positive concept which has gained a lot of attention in the past decade: that of fiscal space [11–14]. “In the broadest sense, ‘fiscal space’ can be defined as the capacity of government to provide additional budgetary resources for a desired purpose without any prejudice to the sustainability of its financial position” [15]. The concept of fiscal space has been refined over time and encompasses a number of policy options, the most commonly used being mobilizing additional revenues, increasing prioritization of budget toward health, and improving technical and allocative efficiency of health expenditure – or a combination of these [16].

In 2019, Benin had an estimated nominal GDP of 1218 USD per capita, and an estimated GDP annual growth (at constant prices) of 6.9% [17]. The International Monetary Fund considers that Benin’s macro-budgetary situation is gradually improving: the authorities are rationalising current expenditure [notably through a wage bill rationalisation], improving the efficiency of public investment, and strengthening the mobilisation of domestic revenue. The 2019 budget provided for an ambitious effort to mobilise tax revenue. Taxes were expected to increase by 1.5% of GDP in 2019, so as to create fiscal space for increased public investment and pro-poor spending [18]. The fiscal deficit actually narrowed significantly in 2019 to 0.5% of GDP, much lower than anticipated, thanks to an over performance of domestic tax and non-tax revenues. With a debt ratio of 41.2% of GDP in 2019, the risk of debt distress remains moderate. Short-term economic prospects have been revised downward because of the Covid-19 shock, but the medium-term outlook continues to be favourable [17].

There is still no clarity as to the funding mechanism of the ARCH itself. An assessment of innovative financing for health was performed in 2015. It was estimated that, among the considered options, taxes on flight boarding and on alcohol would help raise the greatest amount of revenues, and that a combination of five new taxes could enable the government to raise about an additional 54 million USD per year [19] (about 0.36% of GDP). Not all recommendations from this study were implemented however, and it is interesting to note the opacity of the ARCH scheme financing. Apart from the personnel and functioning expenditure of the unit and agency in charge of it, the entire funding of the health insurance pilot project – estimated at 2.86 billion CFA francs (about 4.78 million USD or about 0.03% of GDP) – is entirely off-budget, and based on external contributions. As for the long-term funding of the ARCH when scaled up, according to several interviewed high ranking decision makers from the ARCH project, the Ministry of Health and donors, three innovative taxes were supposed to have been launched in 2016 to finance the ARCH project (on flight boarding, on mobile phone charges, in the form of excise duties) which were supposed to bring in about 9 billion CFA francs (15.1 million USD or about 0.10% of GDP) per annum. Another innovative financing source, entitled a solidarity tax, imposed on voluntary private health insurance premiums, is also planned to complete the scheme in the future. Note first that these taxes are largely insufficient, compared to the expected costs of the premiums to be subsidised by the state – estimated at 20.26 billion CFA francs or 33.90 million USD (about 0.23% of GDP) under the assumptions explained above. However, an examination of the budget law paints another picture: the tax on flight boarding was launched by the previous government in 2013, and increased in 2017; a tax on short text messages was instituted in 2016 (also before the current government was in power) but revoked in 2018. The excise duties have existed for even longer. Moreover, while the interviewed persons seem to believe these taxes were earmarked for financing the ARCH project, actually, the special account created in 2014 to finance the former government’s health insurance scheme (entitled *Régime d’Assurance Maladie Universelle*), was indeed funded by 2 % of the tax on mobile phone charges and 1% of the boarding tax, was suppressed in 2019, and leftover funds were transferred to the general Treasury account [20, 21]. Thus it appears that the resources necessary to finance the ARCH project are not only insufficient, but also far from secured. According to unofficial information communicated during our missions in 2019 and 2020, there was still a financing gap of 241 billion CFA francs (403.2 million USD in total, or about 2.74% of GDP) for the ARCH project as a whole over the period 2019–2023, of which an estimated gap of 108 billion CFA francs (180.7 million USD, i.e., on average, 36.1 million USD or about 0.25% of GDP per year) for the health insurance scheme. The Agency in charge of managing social health insurance has seen its budget reduced from 548 million CFA francs in 2017 – of which 500 million (about 836,500 USD) in the form of subsidies to other entities – to 271 million CFA francs in 2019 – of which only 200 million (about 334,600 USD) in the form of subsidies [22, 23].

Beyond the cost of the social health insurance, it is important that the government continues financing the supply-side elements of the health system. A recent estimation of the additional expenditure needed to achieve the sustainable development goals shows that Benin would have to almost double its health expenditure, and find additional spending equal to 5.1% of GDP in the health sector by 2030, notably to allow the hiring of 8 times more doctors and 4 times more support staff [18].

Overall, Benin faces huge budgetary needs to finance the ARCH social insurance scheme plus the supply-side of the health sector. The tax code in Benin is quite complex and the fiscal policy is continuously evolving [24]. The government has started to implement a number of fiscal measures for that purpose, but they may not all be sufficiently efficient and sustainable, especially since African contexts are characterised by non-linear growth patterns [25]. The above-mentioned specific taxes are certainly not sufficient to sustain funding for the UHC scheme – even in the event that they were actually earmarked for it. Overall, fiscal space for health studies show significant power of economic growth, budget reprioritisation and efficiency improvement measures to drive fiscal space for health expansion, but the limited evidence available is not conclusive in showing potential for earmarked funds, be it in the form of public health taxes or social health insurance contributions, to provide a large-scale, sustained expansion of fiscal space for health [14, 26]. So, while the broad fiscal measures pointed to above are laudable, it appears that the authorities seem to have considered mainly the policy option of mobilizing additional revenues so as to create fiscal space for the ARCH project, but has overlooked the options of increasing prioritization of budget toward health, and improving technical and allocative efficiency of health expenditure. One may question whether the government will continue to allocate sufficient resources from the general Treasury account to fund the ARCH project over the long term. Indeed, the financing scheme must not be subject to annual trade-offs in the framework of the ordinary budgetary process. Moreover, Benin faces a (moderate) risk of over-indebtedness [18], thus possibly compromising aggregate fiscal discipline.

### From resource allocation to strategic purchasing

A second level of desired public sector outcomes expected from the public expenditure management system deals with ensuring that resource allocation and resource use reflects strategic priorities; it stems from the planning function relating to the future allocation of resources [10]. At this level again, this principle has been “modernised” and has attracted a lot of attention in recent years through the promotion of the principle of “strategic purchasing”. Strategic purchasing basically requires switching from reimbursing services or incrementally revising line-item budgets to choosing ex-ante what services better respond to population health needs. It is championed by the World Health Organisation as a key means for health system strengthening on the path to UHC, enabling increased efficiency and value for money, while also improving equity in terms of benefit entitlements and stable healthcare delivery [27–29].

Despite being very fashionable, there is no consensual definition of strategic purchasing, the evidence base is limited, and many possible instruments may be used to render purchasing more strategic [30]. In Benin, the bulk of the healthcare payment mechanisms is done through budget allocation (which is currently done on a historical basis) and fee-for-service payment, and health expenditure is mainly directed towards hospitals (which accounted for nearly 30% of the total health expenditure in 2013, against 20% for preventive services, 18% for medical products, 15% for ambulatory services and 14% for administration) [3, 31], which does not bode well for the strategic purchasing of health services since primary healthcare is by far more the most efficient and equitable level of services to progress towards UHC [1]. Things are not going to improve at this level, since among the budget priorities of the Beninese government for 2019, was the planned construction of five new district hospitals [32].

Among the numerous possible strategic purchasing instruments that may guide the ambitious reforms implemented in Benin, the choice of the service package covered by the ARCH insurance scheme is particularly relevant and timely. Ideally, such a package should be chosen so as to respond to the population’s health needs and preferences. For a number of reasons (e.g. the influence of physicians, whose main professional orientation is curative practice, on policy decisions; personal felt needs for specialised clinical care by the elite decision makers), nascent health insurance systems are often oriented toward curative clinical services [33]. This is also the case in Benin. Despite two actuarial studies having been performed to guide the choice of the ARCH benefit package, the final choice, operated during the first quarter of 2019, resulted from negotiations within the government, and was principally driven by budget constraints. Indeed, while one actuarial study recommended opting for a benefit package corresponding to an insurance premium amounting to about 30 USD per person per annum [34], the chosen package’s estimated cost is only slightly about ten USD per person per annum. The essential benefit package comprises only curative services relative to various disorders (diarrheal diseases, malaria, respiratory infections and other infections in under-five children, dermatological conditions and ear, nose and throat conditions in under-five children, pregnancy and childbirth services, surgical emergencies and trauma) provided along the three levels of the health systems pyramid. This is fine as long as there is no “crowding out” effect between the government’s subsidies to the demand for curative services at the expense of the supply-side financing of the health system, and of services that do not rely on user fees. Indeed, it is important that the government continues financing capital investment, preventive and promotional health services, as well as intersectoral action targeted on the social determinants of health, which are necessary in terms of public health, efficiency and financial sustainability [35–37]. Benin should be inspired by the experience of Ghana, for instance, where the development of the National Health Insurance Scheme – largely financed by earmarked and other public funding – has been accompanied by a reduction in the investment and operational budgets of the Ministry of Health and the Ghana Health Service. This has led in turn to a worrying reduction in operating budgets, including those for the financing of important preventive activities such as immunisation [38]. It has been gauged that because the National Health Insurance Scheme does not explicitly cover preventive services (e.g. check-ups, family planning, malaria prevention), but covers later stages of health issues, it provides weak incentives for cost-effectiveness and drives up claims expenditures [39].

### Operational efficiency

A third level of desired public sector outcomes expected from the public expenditure management system deals with the efficiency and effectiveness of programmes and service delivery resulting from the resource management function [10]. The links between public expenditure management and the health sector are multiple. It is generally admitted that a good public expenditure management system positively impacts on the financing function of the health system and its outcomes, even if evidence is scarce and mitigated [40–42]. The latest Public Expenditure and Financing Accountability evaluation of Benin, dating back to 2014, identifies a number of problems in relation to public expenditure management. It concludes that the efficient provision of services is affected by the insufficiencies noted in the procurement system, and that the common resort to exceptional expenditure procedures does not allow the government to determine the real cost of public services [43]. Since then, the public expenditure management system has been improved in a number of respects [18], but the execution rate of the Ministry of Health’s budget remains low (76.46% on a commitment basis in 2016, but only 44.12% for capital expenditure) [44].

Specifically, health facilities receive financial resources from four broad sources: the State budget (operating budget, public investment programme, and specific programmes), parastatal and private pooled funds (e.g. pension funds, private insurance, community-based insurance), donors and non-governmental organisations, and households through user fees [3]. The efficiency of the expenditure process is probably reduced due to the multiplicity of pooled funds utilised to finance health programmes: a study realised in 2014 identified no fewer than 19 health financing schemes, including five fee exemptions schemes [45]. These mechanisms, which represent 25 to 30% of public health expenditures, use many financing channels; they are not always underpinned by legal provisions; and the purchasing bodies are sometimes also different [3]. The multiplicity of financing channels not only reduces operational efficiency, but also very likely decreases the overall cost-effectiveness of health expenditure, since no comprehensive analysis of the opportunity and effectiveness of expenditure can be made across all the existing budgets. Moreover, one important lesson associated with the unsuccessful experience of performance-based financing in Benin deals with the lack of financial and managerial autonomy on the part of health facilities and even health districts, which prevents health managers taking the appropriate decisions on the right mix of inputs they need to produce results [46].

The ARCH policy aims in the long term to partially unify, or at least make existing financing mechanisms more rational (especially fee exemptions), but it is known from the literature that it is politically difficult to integrate schemes once they are institutionalised because integration involves the redistribution of resources across organised interest groups [47]. Moreover, two other dangers are currently looming and may jeopardise the financial sustainability of health facilities, and hence the operational efficiency and effectiveness of programmes and service delivery: first, the nascent ARCH insurance scheme might encounter teething troubles just as its sister scheme in Ghana did, resulting in the growth in claims expenditures outpacing the growth of insurance revenue, causing a sizable deficit soon transferred to health facilities through long reimbursement delays [39]; second, the Ministry of Finance is considering obliging health facilities to transfer revenues from user fees to a common Treasury account, which would obviously choke health facilities financially, depriving them of the necessary resources to cover daily expenses.

## Discussion

Experience from past reforms promoted by international financial institutions shows that “[t] he pursuit of aggregate fiscal discipline is often done in such a way as to undermine the performance” relative to the two other objectives of a public expenditure management system – “arbitrarily reordering priorities and devastating service delivery and operational performance more generally” [10]. Nowadays, fortunately, the international community is particularly committed to finding “… more money for health, and more health for the money” [48], thus placing emphasis on the allocative and operational efficiency of health expenditure.

Just like many UHC reforms in other countries, the ARCH reform in Benin generates important challenges that go beyond technical issues, and require tricky political choices. The choice made by the government with regard to covering the poor with the use of a specific scheme, intends to reduce inequities in access to health services, but entails a number of risks: pooling risks at too low a level, thus reducing the potential of cross-subsidisation by the richer and healthier populations; maintaining a highly fragmented system, which generates inefficiencies and is difficult to circumvent at a later date [49–51]; developing a two-tier system – especially if budget constraints reinforced by an inefficient ARCH funding system prevent the government from financing a decent service package for the poor; and engendering galloping health costs and reducing the cost-effectiveness of the whole system. Such trade-offs should be assessed and discussed through sufficiently inclusive deliberative processes, in order to ensure sufficient consideration for increased access for all categories of population according to needs, and ownership of reforms by stakeholders [52]. However, our interviews indicate that, whereas the reform of the governance of the supply side of the health system was grounded on a wide consultation process, the model for financing and operationalising the ARCH health insurance project was elaborated by a very limited number of technocrats, without adequate social consultation, whereas donors and non-governmental stakeholders are keen to be involved in policy dialogue. Our inability to get official data on the financing of the off-budget pilot project also testimonies of the opacity of the ARCH management to date, which is deplored by many of our interlocutors. Combined with the failure of the previous government’s attempt to install such a scheme [8], this results in limited ownership and lack of trust in the project by the health sector stakeholders and the general population.

## Conclusion

Benin faces important budgeting challenges with regard to implementing its UHC policy, similar to those also faced by many other African countries. We have highlighted several of them – especially the need to find sufficient, efficient, stable and sustainable resources to finance the social insurance aspect; the need to make sure that the resources dedicated by the government to the social health insurance system are not compensated for by a reduction in capital investments in the sector and/or a reduction in the financing of preventive and promotional primary healthcare services; and the need to improve the efficiency of public expenditure and the financial autonomy of health facilities. In addition, implementation issues further jeopardise the ambitious ARCH project, and will have to be closely monitored so as to facilitate its expansion. As for the scale-up phase of the ARCH scheme, it will be essential to guarantee the overall coherence of the ARCH funding system and to clearly articulate the possible funds from the different sources of financing. Ideally, a common pool should be set up to guarantee cross-subsidies between the different categories of population. The defragmentation of existing schemes is necessary, but will most certainly be politically difficult to implement. At this level too, transparency and negotiation are needed to ensure the political feasibility of the reform.

## Data Availability

Financing data are publicly available on the Global Health Observatory Data Repository. Mission reports (in French) are available on request.

## Abbreviations

ARCH: *Assurance pour le Renforcement du Capital Humain*
CFA: *Communauté française d’Afrique*
GDP: Gross domestic product
UHC: Universal health coverage
USD: Dollar of the United States of America
